# Advances in genetics of migraine

**DOI:** 10.1186/s10194-019-1017-9

**Published:** 2019-06-21

**Authors:** Heidi G. Sutherland, Cassie L. Albury, Lyn R. Griffiths

**Affiliations:** Genomics Research Centre, Institute of Health and Biomedical Innovation. School of Biomedical Sciences, Queensland University of Technology (QUT), Brisbane, QLD Australia

**Keywords:** Migraine, Genetics, Migraine without aura, Migraine with aura, Hemiplegic migraine, Mutation, Variant, Single nucleotide polymorphism, Genome-wide association study

## Abstract

**Background:**

Migraine is a complex neurovascular disorder with a strong genetic component. There are rare monogenic forms of migraine, as well as more common polygenic forms; research into the genes involved in both types has provided insights into the many contributing genetic factors. This review summarises advances that have been made in the knowledge and understanding of the genes and genetic variations implicated in migraine etiology.

**Findings:**

Migraine is characterised into two main types, migraine without aura (MO) and migraine with aura (MA). Hemiplegic migraine is a rare monogenic MA subtype caused by mutations in three main genes - *CACNA1A*, *ATP1A2* and *SCN1A* - which encode ion channel and transport proteins. Functional studies in cellular and animal models show that, in general, mutations result in impaired glutamatergic neurotransmission and cortical hyperexcitability, which make the brain more susceptible to cortical spreading depression, a phenomenon thought to coincide with aura symptoms. Variants in other genes encoding ion channels and solute carriers, or with roles in regulating neurotransmitters at neuronal synapses, or in vascular function, can also cause monogenic migraine, hemiplegic migraine and related disorders with overlapping symptoms. Next-generation sequencing will accelerate the finding of new potentially causal variants and genes, with high-throughput bioinformatics analysis methods and functional analysis pipelines important in prioritising, confirming and understanding the mechanisms of disease-causing variants.

With respect to common migraine forms, large genome-wide association studies (GWAS) have greatly expanded our knowledge of the genes involved, emphasizing the role of both neuronal and vascular pathways. Dissecting the genetic architecture of migraine leads to greater understanding of what underpins relationships between subtypes and comorbid disorders, and may have utility in diagnosis or tailoring treatments. Further work is required to identify causal polymorphisms and the mechanism of their effect, and studies of gene expression and epigenetic factors will help bridge the genetics with migraine pathophysiology.

**Conclusions:**

The complexity of migraine disorders is mirrored by their genetic complexity. A comprehensive knowledge of the genetic factors underpinning migraine will lead to improved understanding of molecular mechanisms and pathogenesis, to enable better diagnosis and treatments for migraine sufferers.

## Background

### Migraine types and classification

Migraine is a common type of primary headache disorder, distinguished by recurrent attacks of moderate to severe unilateral throbbing pain, often accompanied by nausea and/or photophobia and phonophobia. It is classified into two major types: migraine without aura (MO) and migraine with aura (MA), with visual, sensory or other central nervous system (CNS) symptoms preceding the headache and associated migraine symptoms, in the latter [1]. Other subtypes or forms have been classified, including chronic migraine and episodic syndromes associated with migraine. Hemiplegic migraine (HM) is a rare, severe subtype of MA, in which migraine symptoms are accompanied by motor symptoms such as temporary numbness or weakness, affecting one side of the body (hemiparesis). Familial hemiplegic migraine (FHM) is a familial form of HM where it is usually inherited in an autosomal dominant manner. Investigating the genetic basis of FHM, as well as the common types of MO and MA, has greatly helped in our understanding of migraine pathophysiology through the discovery of the genes that contribute to the disorder.

### Migraine phases and pathophysiology

#### Activation of the trigeminovascular system

Migraine is thought to be a complex brain network disorder that occurs when the brain loses control of its homeostasis, leading to the activation of the trigeminovascular system and a cascade of events [2]. Signals from activated nociceptors innervating the cranial blood vessels are transmitted to the trigeminal bipolar neurons, and further relayed to thalamic and cortical areas [3, 4]. The signal from the perivascular neurons is transmitted by endogenous mediators, including the vasoactive neuropeptides calcitonin gene-related peptide (CGRP), substance P, neurokinin A, and pituitary adenylate cyclase-activating peptide (PACAP), as well as release of vasoactive inflammatory mediators such as nitric oxide, coincident with inflammation in the meninges [2, 5]. Sensitization of pain relevant brainstem regions, including peripheral trigeminovascular neurons to dural stimuli, is thought to produce the characteristic sensation of throbbing pain in migraine [6, 7].

#### Migraine progression and mechanisms

During migraine, distinct areas of the brain are activated, each contributing to aspects of migraine pathophysiology, whether this is triggering the attack, generating the pain, or playing roles in some of the associated neurological symptoms that occur during an attack [2]. Migraine is characterised by multiple phases; trigeminal activation occurs in the headache phase, but these may be preceded by a premonitory phase, in which symptoms including fatigue, mood changes, food cravings, yawning, muscle tenderness, and photophobia may be experienced up to 3 days before the headache [8]. Some individuals also experience an aura phase, which may feature visual, sensory, speech/language, and motor disturbances, as well as disruption of higher cortical function, immediately preceding or concurrent with the headache [8]. Cortical spreading depression (CSD) is a slowly propagating wave of depolarization in neuronal and glial cell membranes accompanied by massive ion fluxes, which spreads across the brain cortex, followed by a suppression of activity [9]. It coincides with the initiation and progression of aura symptoms, but whether CSD is causally linked to the initiation of headache is still debated [10]. Evidence from experimental animals supports a pivotal role of CSD in aura, headache initiation and activation of trigeminal nociception [11–13]; CSD-associated opening of neuronal Panx1 mega-channels releases molecules that trigger an inflammatory cascade, which activates neighboring astrocytes and leads to sustained release of inflammatory mediators [13]. Most migraineurs, however, do not experience aura, and it is unlikely that CSD is involved in initiating the complete syndrome of migraine. Alternative triggers for trigeminovascular activation, such as cortical hyperexcitability and brain stem or hypothalamic dysfunction, may also be important [14].

#### Brain alterations in migraine

A variety of imaging techniques have revealed both structural and functional brain alterations in individuals that suffer migraine [14]. Furthermore, clinical and neurophysiological studies have found chronic hypersensitivity to sensory stimuli and or abnormal processing of sensory information in migraineurs [15–17], as well as cortical excitability which may make them more susceptible to CSD [17, 18]. While some of these changes may be the result of repetitive exposure to pain or stress, the brain biology of migraine sufferers appears to differ from healthy controls [2]. Migraine may be triggered by a range of external factors, including chemicals, lack of sleep, stress, and skipping meals. However, these triggers only lead to migraine in migraineurs. Some aspects of the altered brain biology are likely to be genetically predetermined.

### A genetic basis for migraine

Family and twin studies have demonstrated that there are genetic factors that contribute to the susceptibility of an individual to migraine. This is clear for individuals with monogenic migraine disorders, such as FHM, in which a pathogenic variant in a single gene can lead to the disorder, with nearly complete penetrance. Family and twin studies also suggest that common migraine is also a heritable trait, with heritability estimated between 30 and 60% [19–21]. Common migraine forms, including MO and MA, are most likely due to the contribution of variants with small effect at many genetic loci, i.e. these are considered to be polygenic disorders. Different approaches have been used to identify and understand the function of the genes involved in monogenic and polygenic migraine. For the former, this has been achieved by linkage mapping of genetic markers and sequencing of candidate genes in family pedigrees featuring the disorder, followed with functional studies in cellular and animal models. In recent years Next-generation sequencing (NGS) techniques have accelerated the discovery of genes and variants linked to monogenic migraine-related disorders. With regards to polygenic forms, genome-wide association studies (GWAS) in large migraine case-control cohorts has greatly helped our understanding of the many genetic factors and pathways that contribute to common migraine, with subsequent transcriptomics and functional experiments required for further understanding of the causal mechanisms.

## Main text

### Genetics of monogenic migraine disorders

Valuable insights into how some of the underlying genetic factors contribute to the pathophysiology of migraine have been provided by a number of rare inherited migraine disorders, which can be caused by mutations in a single gene (Table 1). These include hemiplegic migraine (HM) and familial migraine (where migraine is inherited in a Mendelian manner), as well as a range of monogenic neurological and vascular disorders which can show symptomatic crossover. The latter include some types of episodic ataxias, paroxysmal movement disorders, and the stroke syndrome cerebral autosomal dominant arteriopathy with subcortical infarcts and leucoencephalopathy (CADASIL; Mendelian Inheritance in Man catalogue, MIM #125310), and commonly feature migraine and/or episodic attacks of associated symptoms such as motor weakness, vertigo and nausea, along with their other characteristic symptoms.

**Table 1:** Migraine-related monogenic neurological and vascular disorders and their causal genes and mechanism of mutations

Disorder	Symptoms/Key Clinical Findings	Causal gene/s	Mutations and mechanisms
With mostly neurological symptoms and signs:			
Familial Hemiplegic Migraine (FHM)	• Migraine • Visual disturbances • Motor weakness (e.g. hemiplegia, ataxia, nystagmus) • Sensory Loss (e.g. numbness) • Difficulty with speech (e.g. dysphasia, aphasia) • Additional neurological symptoms (e.g. confusion, seizures, memory loss, coma)	*CACNA1A*	*Missense, gain-of-function ↑ Ca* ^*2+*^ *influx into the presynaptic terminal resulting in excessive neurotransmission.*
		*ATP1A2*	*Missense, partial-to-complete loss-of- function ↑synaptic K* ^*+*^ *and glutamate triggering neuronal hyperexcitability.*
		*SCN1A*	*Spectrum of FHM3 mutations is highly complex, biological mechanisms remain unclear – however mutations result in a ↓ in inhibitory transmission which triggers a ↑ in excitatory transmission.*
Mendelian Migraine with Aura	• Typical Migraine with Aura	*KCNK18*	*Frameshift mutation in TRESK potassium channnel, but has dominant negative effect due to alternatively translated TRESK fragment which downregulates TREK1 and TREK2 potassium channels.*
Episodic Ataxia type 2 (EA2)	• Migraine • Nystagmus • Muscle weakness • Paraesthesia • Progressive cerebellar ataxia (e.g. imbalance and incoordination) • Vertigo	*CACNA1A*	*Loss-of-function mutations result in decreased channel function and thereby a ↓ in intracellular Ca* ^*2+*^ *; how this triggers EA2 disease mechanisms remains unclear.*
Spinocerebellar Ataxia type 6 (SCA6)	• Migraine • Cerebellar atrophy • Dysarthria • Nystagmus • Progressive cerebellar ataxia • Sensory neuropathy (e.g. pins and needles, tingling and burning)	*CACNA1A*	*Expansion of ‘CAG’ polyglutamine repeats in COOH tail of CACNA1A protein, toxic gain-of-function affecting channel regulation s → selective degeneration of cerebellar Purkinje cells*
Familial Advanced Sleep-Phase Syndrome (FASPS) 2	• Disrupted circadian rhythms (e.g. early onset and offset sleep-wake cycles) • Migraine with Aura	*CSNK1D*	*Loss-of-function (partial). Casein kinase Iδ phosphorylates mammalian clock protein PER2. CKIδ also phosphorylates and regulates GJA1/Connexin43, an astrocytic gap junction protein and a migraine GWAS loci.*
ROSAH syndrome	• Ocular (e.g. retinal dystrophy, optic nerve edema, low-grade inflammation), • Splenomegaly • Anhidrosis • Migraine headache	*ALPK1*	*Possible gain-of-function. May affect ciliary formation, regulation of apical transport. Etiology of migraine unclear, but kinase function may affect CGRP activity.*
Paroxysmal dyskinesia disorders	• Recurrent and brief attacks of involuntary movement (can be induced by voluntary movements, [PKD], coffee, alcohol, strong emotion [PNKD] or exercise [PED] • Can present with, or have, accompanying hemiplegic migraine	*PRRT2*	*Missense or most frequently loss of function truncating mutations in PRRT2, result in ↑ presynaptic vesicle release and excitatory transmission, possibly a modifier gene for hemiplegic migraine.*
		*PNKD*	*Missense mutations affect protein cleavage and stability. PNKD interacts with synaptic active zone proteins and mutant protein is less effective at inhibiting exocytosis, resulting in ↑ neurotransmitter release. *
		*SLC1A3*	*Spectrum of FHM3 mutations is highly complex, biological mechanisms remain unclear – however mutations result in a ↓ in inhibitory transmission which triggers a ↑ in excitatory transmission.*
With mostly vascular symptoms and signs:			
Cerebral Autosomal Dominant Arteriopathy Subcortical Infarcts with Leukoencephalopathy (CADASIL)	• Premature stroke • Cognitive disturbances (e.g. dementia, • psychiatric issues varying from personality changes to severe depression, coma, confusion) • Difficulty with speech (e.g. aphasia) • Motor weakness (e.g. hemiplegia) • Migraine with aura • Seizures	*NOTCH3*	*Usually gain-of-function cysteine residue mutations, which produces toxic NOTCH3 protein accumulation and progressive damage in neuronal blood vessels.*
COL4A1/A2 disorders	• Stroke and small vessel disease (e.g. porencephaly, leukodystrophy • Eye defects (e.g.retinal arterial tortuosity, Axenfeld-Rieger anomaly, cataract) • Systemic effects (e.g. kidney, muscle cramps, Raynaud phenomenon, cardiac arrhythmia, and hemolytic anemia • Migraine with and without Aura	*COL4A1* *COL4A2*	*Usually missense mutations of highly conserved glycine residues in the Gly-X-Y repeat of the collagen triple-helical domain, which impair collagen IV heterotrimer assembly. Some truncating mutations resulting in haploinsufficiency.*
Retinal vasculopathy with cerebral leukodystophy (RVCL)	• Vascular retinopathy, visual loss • Mini-strokes, cerebral leukodystrophy • Cognitive disturbances (e.g. depression, seizures, mental impairment) • Migraine (mainly without aura) • Mild renal and liver dysfunction • Raynaud’s phenomenon and gastro- • intestinal bleeding in some individuals	*TREX1*	*C-terminal truncations of TREX1 3′-5′ exonuclease which result in its mislocalisation in the cell, which causes dysregulation of the ER oligosaccharyltransferase (OST), release of free glycans and potentially glycosylation defects.*

### Hemiplegic migraine

Hemiplegic Migraine (HM) is a rare subtype of MA characterised by episodes of severe migraine and aura symptoms involving motor weakness or numbness, usually affecting one side of the body (hemiparesis), as well as visual, sensory, or speech disturbances [1, 22]. In some cases, patients experience additional neurological symptoms such as confusion, seizures, memory loss, and coma. Individuals usually fully recover between episodes, although some symptoms may persist for weeks or longer, and some patients can develop permanent ataxia (difficulty coordinating movements), which may worsen with time [23]. In rare cases HM can be fatal after a minor head trauma [24].

#### Familial hemiplegic migraine (FHM)

The prevalence of HM has been found to be up to 0.01% in European populations, with both familial and sporadic forms [23, 25, 26]. FHM is diagnosed when there is at least one 1st or 2nd degree relative in the family who also suffers HM attacks. FHM usually shows an autosomal dominant pattern of inheritance (with 70–90% penetrance) and is considered to be monogenic, but genetically heterogeneous. To date three main causative genes – *CACNA1A*, *ATP1A2* and *SCN1A* – have been identified through linkage studies and mutational screening in FHM family pedigrees. FHM can be classified as FHM1 (MIM #141500), FHM2 (MIM #602481) and FHM3 (MIM #609634) according to whether patients have mutations in *CACNA1A*, *ATP1A2* or *SCN1A*, respectively. Clinically these FHM sub-types are indistinguishable, as symptoms overlap, but there is wide variation in phenotypes, including between individuals with mutations in the same gene, or even family members with the same mutation [27–29]. This suggests that other genes or environmental factors can modify phenotype. It should be noted that the majority of cases (< 25%) do not appear to have mutations in the *CACNA1A*, *ATP1A2* or *SCN1A* genes [30] and our results (under review). Nevertheless, identifying and studying the known FHM genes and mutations has greatly improved diagnostics as well as understanding of the underlying biology of HM. The three main HM genes encode ion channel or ion transport proteins, leading to the supposition that HM is a channelopathy [31].

##### FHM1 due to mutations in CACNA1A

*CACNA1A* on chromosome 19p13 was the first gene implicated in FHM (FHM1), identified via positional cloning and mutation analysis of candidate genes in multiple FHM family pedigrees [32]. The gene encodes the pore-forming α1 subunit of the neuronal voltage-gated Ca_v_2.1 (P/Q-type) channels, which are predominantly localized at the presynaptic terminals of brain and cerebellar neurons, and play an important role in controlling neurotransmitter release [33]. > 25 pathogenic variants in *CACNA1A* have been reported for FHM1, which are inherited in an autosomal dominant pattern. *CACNA1A* deletions have been reported in FHM1 patients [34, 35], however the majority are missense variants, lying in significant functional domains of the calcium channel, i.e. the voltage sensor, pore, and pore-lining loops [36]. They usually have gain-of-function effects, leading to increased Ca^2+^ influx, which results in enhanced glutamatergic neurotransmission and neuronal hyperexcitability [32, 37, 38]. While a strict genotype-phenotype correlation does not exist [29, 39], symptoms and clinical severity may vary depending on the variant [40, 41]. Transgenic FHM1 knock-in (KI) mouse models have been generated: one, which expresses the milder R192Q *CACNA1A* mutation, shows no overt phenotype [42], while another with the severe S218 L mutation exhibits cerebellar ataxia and spontaneous seizures in accordance with severity of the clinical symptoms observed in patients (28). In both these mouse models the FHM1 mutations cause gain-of-function effects, leading to altered cortical excitatory-inhibitory balance, increased neurotransmission, and increased susceptibility to CSD action [42–45]. Additionally, increased trigeminal sensory firing [44, 46, 47], tissue anoxia attributing to prolonged aura [48], head pain when triggered [49], and altered CGRP-mediated trigeminal pain signalling and synaptic plasticity [4, 50], have been observed in FHM KI models.

What controls trigeminal sensory excitability in between FHM attacks remains unknown [44]; this, in conjunction with extreme clinical diversity and variability, suggests that a number of environmental factors and/or modifier genes may act independently on the function of neuronal P/Q calcium channels as compensatory mechanisms until a threshold is reached [29]. Screens for genetic modifiers in animal models are consistent with this. For example, genetic knockdown of Drosophila phospholipase C beta (PLCβ, which is involved in cardiovascular and neuronal signalling), or genetic variants affecting the receptors that gate intracellular calcium stores (e.g. inositol triphosphate [IP3] and Ryanodine receptors), partially alleviated some of the electrophysiological phenotypes of FHM1 mutations [51]. In another example, a large-scale functional RNAi screen in *Caenorhabditis elegans* for modifiers of *unc-2*, the worm orthologue of *CACNA1A*, identified genes in the TGF-β and Notch signalling pathways [52]. Interestingly, those pathways are relevant to both common migraine, as revealed by association studies [53], as well as other monogenic disorders such as CADASIL which has overlapping symptoms with FHM [54]. Studies in FHM1 transgenic mice have also demonstrated the role of female sex hormones in increased susceptibility to CSD (37), suggesting that hormones are also modifying factors, and may explain some of the variable expressivity and penetrance of FHM pathogenic variants and the female preponderance of migraine disorders (49).

##### Episodic Ataxia 2 and spinocerebellar Ataxia type 6 due to mutations in CACNA1A

In addition to FHM1, heterozygous mutations within *CACNA1A* can cause two other neurological disorders, episodic ataxia type 2 (EA2; MIM #108500) and spinocerebellar ataxia type 6 (SCA6; #MIM 183086) [32, 55]. EA2 is characterized by paroxysmal attacks of ataxia, vertigo, and nausea, while SCA6 is typified by adult-onset, slowly progressive cerebellar ataxia, dysarthria, and nystagmus. There can be overlapping clinical features between the three allelic disorders [56], e.g. ~ 50% EA2 patients also suffer migraine [57], and episodic headaches and nausea are also common in SCA6 [58]. EA2 mutations can be missense, truncating or cause aberrant splicing of *CACNA1A* [59]. However, unlike FHM mutations, they are usually loss-of-function and result in decreased Ca^2+^ influx [4]. SCA6 mutations are usually small expansions of a polyglutamine repeat in the COOH tail of *CACNA1A* [55] which leads to accumulation of mutant Ca_v_2.1 channels and selective degeneration of cerebellar Purkinje cells due to a toxic gain-of-function effect [60].

##### FHM2 due to mutations in ATP1A2

In 2003, *ATP1A2* at 1q23.2 was identified as second major FHM gene [61]. *ATP1A2* encodes the α2 isoform of the catalytic subunit of the Na^+^/K^+^-ATPase ion transport pump, which is responsible for regulating electrochemical gradients across the cell membranes of the CNS, heart, skeletal and smooth muscle tissue [62]. The pump is mainly expressed on astrocytes at tripartite synapses in the CNS, and its function in the clearance of extracellular K^+^ and production of a Na^+^ gradient used in the reuptake of glutamate, is important to its role in HM [63]. *ATP1A2* mutations (FHM2) are usually inherited in an autosomal dominant pattern, and patients have a wide clinical spectrum [62, 64], which includes neurological disorders such as alternating hemiplegia of childhood [65], epilepsy [66], seizures [67], and permanent mental retardation [68, 69], as well as neuromuscular periodic paralysis disorders [70] and recurrent coma and fever [71], secondary to recurrent FHM-like attacks. > 80 causal variants have been linked to FHM2, with ~ 25 diagnosed in sporadic cases, suggesting that de novo mutations are common at the *ATP1A2* locus [62]. While *CACNA1A* mutations are reported as the most common in some HM cohorts [36, 72], using an NGS panel to screen the three main HM genes in an Australian patient cohort we found that ~two-thirds of the HM mutations identified were in *ATP1A2* (under review).

The majority of FHM2 mutations are missense and cluster in the catalytic P domain, the transmembrane domain, or in the central region between these; small deletions, a mutation causing protein extension through stop codon alterations, and exonic duplication have also been reported [62, 73–75]. In vitro functional models have been used to determine the functional consequences of a number of *ATP1A2* FHM2 mutations, with studies demonstrating significant protein dysfunction ranging from partial to complete loss [62]. *ATP1A2* mutations have been found to: i) alter (increase or decrease) pump sensitivity to potassium [76, 77]; ii) reduce the sodium/potassium turnover rate [40]; or iii) generate non-functional proteins [78–80]. Homozygous Atp1a2 knock-out (KO) mice die immediately after birth [81], and recently biallelic loss of function variants in *ATP1A2* have been reported in humans, resulting in death neonatally, with features of hydrops fetalis, microcephaly, arthrogryposis and extensive cortical malformations [82]. Heterozygote KO mice have altered behaviour and neurological defects [81], but also exhibit a low threshold for induction of CSD, faster propagation rate, and delayed recovery from mass depolarization compared to wild-type mice [83]. FHM2 KI mice carrying either the human W887R or G301R mutations, show altered CSD, with the former more susceptible to CSD due to a reduced rate of glutamate and K^+^ clearance by cortical astrocytes [84, 85], and the latter displaying a prolonged recovery phase following CSD [86]. Therefore, *ATP1A2* mutations have been hypothesised to contribute to FHM pathophysiology by increasing the propensity for CSD action due to increased levels of synaptic K^+^ and glutamate as a result of dysfunctional Na^+^/K^+^ ATPase pump action [87, 88]. While many FHM2 *ATP1A2* mutations abolish or greatly reduce pump activity, others cause more subtle effects, including shifts in voltage dependence, kinetics, or apparent cation affinities [62]. Nevertheless, they affect glutamatergic neurotransmission, causing the defective regulation of the balance of excitation and inhibition in the brain seen in migraine [89].

##### FHM3 due to mutations in SCN1A

*SCN1A* (chr 2q24.3) was identified as a third causative gene for FHM in 2005 [90]. FHM3 is rarer than FHM1 and 2 (up to ~ 10% of patients with a molecular diagnosis). *SCN1A* encodes the α1 subunit of the neuronal voltage-gated sodium channel Na_v_1.1, which mediates the voltage-dependent sodium ion permeability of excitable membranes (primarily the inhibitory gamma-Aminobutyric acid [GABA]-ergic interneurons) of the CNS [91]. *SCN1A* is commonly mutated in epilepsy syndromes with hundreds of heterozygous truncating and missense mutations reported [92]. Eleven FHM3 *SCN1A* mutations have been described to date, and are usually inherited in an autosomal dominant manner [93–95]. Mutations have been identified in both pure FHM families, and also in those with FHM and additional neurological disorders, including generalised tonic-clonic epilepsy, elicited repetitive transient daily blindness and childhood epilepsy [96–98].

Epileptic mutations mainly cause loss-of-function, resulting in reduced sodium currents and action potential firing in GABAergic inhibitory interneurons [99–101]; *SCN1A* KO mice suffer from ataxia and epileptic seizures [102, 103]. In FHM3, mutations in *SCN1A* are usually missense and cause gain-of-function effects on the channel, displaying increased threshold-near persistent current, delayed entry into inactivation, and a faster recovery and higher channel availability during repetitive stimulation [104–107]. This predicts increased firing of inhibitory GABAergic neurons, leading to higher extracellular potassium concentrations, enhanced glutamate release, and triggering of CSD [106, 108]. However, the mechanisms of *SCN1A* mutations in FHM3 can be complicated: some exhibit loss-of-function effects in heterologous cell systems [109]; a *SCN1A* T1174S mutation reported in a family with both epileptic and FHM phenotypes can act in both a gain- and loss-of-function manner [105]; and furthermore, the *SCN1A* L1670 W and L1649Q mutations induce folding and trafficking defects which, when rescued by incubation at lower temperatures, or when expressed in GABAergic cortical neurons, modifies the gating properties leading to an overall gain-of-function [110, 111]. KI mouse models of FHM3 mutations have not been reported to date, but would help further understanding of their mechanisms of pathogenesis.

#### Sporadic hemiplegic migraine (SHM)

Sporadic Hemiplegic Migraine (SHM) is diagnosed when there is no family history of HM, and estimates suggest in the general population approximately one-third of cases are sporadic [25]. SHM can be caused by pathogenic variants in the known FHM genes, including those that have arisen de novo, which may then become familial cases [41, 74, 112] Variants in *ATP1A2* have been the most commonly found in SHM cases, possibly reflecting greater genetic heterogeneity, or more variable penetrance, in this gene [62]. SHM may result from less penetrant variants in the known FHM genes, mosaicism in the transmitting parent, pathogenic variants in other genes, and/or other modes of inheritance, e.g. compound recessive mutations and gene/environment interactions [23, 93]. Some SHM cases may also represent a phenotypic extreme of common migraine due to a combination of lower-risk genetic variants. For example, Pelzer et al. (2018) found that individuals with HM, but without mutations in *CACNA1A*, *ATP1A2* or *SCN1A*, generally have a milder phenotype than those with mutations in those genes [41].

#### Hemiplegic migraine and disorders with overlapping symptoms caused by mutations in other genes

Although rare, pathogenic variants in other genes, including *PRRT2*, *PNKD*, *SLC2A1*, *SLC1A3*, *SLC4A4*, have been reported in HM. Mutations in *PRRT2* and *PNKD* are more commonly associated with paroxysmal conditions, in particular movement disorders [113]. *PNKD* is the main causal gene for paroxysmal non-kinesigenic dyskinesia (PNKD; MIM #118800) [114, 115], while *PRRT2* mutations can cause paroxysmal kinesigenic dyskinesia (PKD; MIM #128200) [116, 117], paroxysmal non-kinesigenic dyskinesia (PNKD) [118], paroxysmal exercise-induced dyskinesia (PED), and childhood epilepsy/seizure disorders [119, 120]. Some patients presenting with HM have been found to have mutations in *PRRT2* [118, 121–124], leading to the suggestion that it is a fourth HM gene [121]. However, the relationship is complicated due to the clinical heterogeneity and pleiotropy of phenotypes, and it may mainly act in a modifying role [125]. *PRRT2* encodes Proline Rich Transmembrane Protein 2 (PRRT2), a presynaptic transmembrane protein which interacts with members of the SNAP Receptor (SNARE) complex [126]. It is involved in synaptic vesicle fusion and regulation of voltage-gated calcium channels in glutamatergic neurons, and is important in the final steps of neurotransmitter release [127–129]. Heterozygous *PRRT2* c.649dupC (p.Arg217Profs*8) or c.649delC (p.Arg217Glufs*12) loss-of-function truncating mutations are the most common in PRRT2-related conditions, including HM, and are likely to result in impaired interaction with the SNAP25/SNARE complex and increased presynaptic vesicle release, leading to a state of hyperexcitability [118].

Mutations in both *PNKD*, the main causal gene for PNKD, and *SLC2A1*, the glucose transporter protein type 1 (GLUT1 or EAAT2) gene implicated in PED and GLUT1 deficiency syndrome (MIM #606777), have also been found in HM patients [118, 130, 131]. They likely act via disruption of neurotransmitter regulation and impaired synaptic vesicle release [118]. Mutations in *SLC1A3*, the gene for the glial glutamate transporter EAAT1, can cause episodic ataxia, type 6, (EA6; MIM #612656), but have also been associated with HM [132, 133]. Similarly, mutations in *SLC4A4*, the gene for the sodium bicarbonate cotransporter NBCe1, which is usually involved in renal tubular acidosis syndromes (MIM #604278) are also found in some HM cases [134]. Analysis of whole exome sequencing (WES) data of HM patients without *CACNA1A*, *ATP1A2* and *SCN1A* mutations suggests that mutations in all these genes are rare [41] and our results (under review), but should be considered in the molecular diagnosis of patients without mutations in the main HM genes.

### Familial migraine with Aura and associated disorders

The majority of studies of migraine in family pedigrees with Mendelian inheritance have focussed on those with the HM phenotype. However, a few cases of familial MA have been reported, which have revealed other genes and molecular mechanisms involved in migraine biology.

#### Familial migraine with Aura caused by mutations in *KCNK18* encoding the TRESK channel

A monogenic form of typical MA in a large multigenerational pedigree identified a frameshift mutation (F139Wfsx24) in the TWIK-related spinal cord potassium channel (TRESK, encoded by *KCNK18*), segregating with migraine [135]. TRESK is a member of the two pore domain potassium channel (K2P) family, which regulate the excitability of a variety of neurons involved in transducing pain stimuli, including the somatosensory neurons of the dorsal root ganglia (DRG) and trigeminal ganglia [136, 137]. KO mouse models suggest TRESK functions to modify certain forms of nociceptive afferentation [138, 139]. Functional analysis suggested a dominant negative effect of the TRESK F139Wfsx24 mutation on whole-cell TRESK currents resulting in hyperexcitability of trigeminal ganglion neurons [140]. However, another dominant negative TRESK mutation, C110R, which is not associated with migraine [141], does not trigger sensory neuron hyperexcitability, even though it reduces TRESK currents in sensory neurons [142]. A recent study by Royal et al. (2019) sheds light on this apparent contradiction and has revealed a novel mechanism by which frameshift mutations can alter the function of a gene [143]. Firstly, they found that TRESK can heterodimerise with two other K2P channels, TREK1 and TREK2, which when knocked out together in mice results in a migraine-like allodynia phenotype. The TRESK-C110R protein inhibits TRESK activity on dimerization, but does not affect TREK1 and TREK2, while TRESK-F139Wfsx24 inhibits activity of all three channels. Interestingly, the 2 bp frameshift puts an alternative start codon in frame, which results in translation of a second TRESK fragment. It is this that specifically downregulates TREK1 and TREK2 function, which appears to contribute to migraine induction. Furthermore, Royal et al. (2019) identified another TRESK frameshift mutation (Y121LfsX44) in a human exome sequence database, and which is associated with migraine in ClinVar, that appears to work via the same mechanism which they have termed frameshift mutation-induced alternative translation initiation [143]. Finally, this work suggests that TREK-related genes may also be involved in migraine.

#### Familial advanced sleep-phase syndrome (FASPS) and migraine caused mutations in *CSNK1D*

Casein kinase 1 delta (CKIδ) is a central component of the circadian clock. Mutations in the CKIδ gene, *CSNK1D*, were found to cause familial advanced sleep phase syndrome (FASPS) in two large independent pedigrees [144, 145]. FASPS patients show severe disruption of the sleep-wake-cycle and other circadian rhythms, but interestingly, the phenotype also co-segregated with MA in these pedigrees. Mice carrying a transgene with the human CKIδ-T44A mutation displayed sensitization to pain after triggering migraine with nitroglycerin, and a reduced threshold for CSD; cultured astrocytes showed increased spontaneous and induced calcium signalling [144, 145]. Further details of its role in migraine are to be elucidated, but CKIδ is a ubiquitous serine-threonine kinase that phosphorylates the circadian clock protein PER2, as well as other proteins involved in brain signalling [146]. *CSNK1D* is a notable exception to the ion channel and glutamatergic-related genes implicated in the majority of monogenic migraine, and the connection between migraine and FASPs is consistent with a likely role of the hypothalamus in regulating physiological stresses and migraine susceptibility [147–149].

#### ROSAH syndrome – retinal dystrophy, optic nerve edema, splenomegaly, anhidrosis and migraine headache – caused by mutations in *ALPK1*

ROSAH is a recently described distinct autosomal dominant ocular systemic disorder, which features migraine headache as one of the key clinical features. Exome and genome sequencing identified a heterozygous missense pathogenic variant in the *ALPK1* gene (c.710C > T, p.[Thr237Met]) in five independent families [150]. *ALPK1* encodes Alpha Kinase 1, which may play roles in inflammation and intracellular trafficking, although its function is poorly defined, and it is not yet understood the how mutations in the protein would contribute to migraine.

### Monogenic vascular disorders which feature migraine

#### Cerebral autosomal dominant arteriopathy with subcortical infarcts and leukoencephalopathy (CADASIL)

There are a number of primarily vascular disorders caused by mutations in single genes, in which migraine is a common symptom. Cerebral autosomal dominant arteriopathy with subcortical infarcts and leukoencephalopathy (CADASIL), is a cerebral small-vessel disease (SVD) characterised by vascular degeneration, recurrent subcortical ischaemic strokes, cognitive decline, dementia, and premature death [54]. It is the most common heritable cause of stroke and vascular dementia in adults, caused by toxic gain-mutations in *NOTCH3*, which are usually autosomal dominant. Migraine, in particular the MA subtype, is a common symptom accompanying CADASIL (in up to 75% cases) [151–154], often presenting decades before the onset of other symptoms [54, 155]. For example, a study of 300 symptomatic CADASIL patients found that three quarters had migraine (90% of which was MA), and in two-thirds of the patients it was the presenting symptom [153].

#### Retinal vasculopathy with cerebral leukodystrophy (RVCL) and *COL4A1*-related SVDs

Other SVDs that commonly feature migraine include syndromes such as retinal vasculopathy with cerebral leukodystrophy (RVCL; MIM #192315) caused by mutations in *TREX1* [156, 157], and *COL4A1* and *COL4A2*-related disorders [158–160]. The exact mechanism through which vascular disorders lead to an increased prevalence of migraine is unknown [154], but they indicate that some genes with roles in vascular function are also implicated in migraine, something which has also become apparent in polygenic migraine from both epidemiological studies and GWAS [161, 162].

### Methods and applications for identifying disease-causing variants in monogenic migraine and related disorders

#### Next-generation sequencing for molecular testing of hemiplegic migraine

Until relatively recently HM genetic testing involved Sanger sequencing of selected exons in one, two or all three main HM causative genes (*CACNA1A*, *ATP1A2* and *SCN1A*). This form of iterative testing was limited and could be costly and time consuming. The development of next-generation sequencing (NGS) technologies, in which millions of small fragments of DNA are sequenced in parallel, have revolutionised genomic research, allowing specific regions of interest to the entire genome to be sequenced concurrently. NGS applications include targeted gene panels, WES (in which all the coding regions of the genome are sequenced), and Whole Genome Sequencing (WGS), which also captures introns, regulatory regions and all other non-coding DNA. NGS has been applied clinically in genetic diagnostics, including for HM and overlapping disorders, facilitating the discovery of novel HM mutations [163–165]. Using a five gene panel designed for HM and overlapping disorders (EA2 and CADASIL), our laboratory has found that diagnostic success rates have increased considerably (~ 21%) when compared to that of previous Sanger sequencing testing methods (~ 9%), and have identified a number of novel causative variants for HM and related disorders [166, 167]. Clinicians also appreciate the option to test for overlapping neurological disorders when presented with complex cases with HM-related symptoms.

#### Discovering new genes in migraine-related disorders

Importantly, recent application of NGS sequencing techniques to screen HM patients have shown that the majority do not have exonic mutations in the main HM genes [30]. We find that > 75% patients sent for testing do not have likely pathogenic exonic variants in *CACNA1A*, *ATP1A2* or *SCN1A* (under review). Furthermore, analysis of data from NGS panels or WES has revealed that likely pathogenic variants in other known familial migraine and migraine-related genes are also rare [41], [our results (under review)]. This low level of diagnostic success may largely be due to other causative genes or genetic factors, although no other major HM loci have been found so far [41]. In addition to the three main genes, HM may be highly genetically heterogeneous. From what is already known of the biology, other genes likely to be involved in HM may include ion channel and solute transporter genes, as well as genes involved in aspects of glutamatergic neurotransmission and vascular biology. Assigning causality for variants that are less dominant or penetrant than those in the known HM genes will be challenging. This is exemplified in a study by Klassen et al. (2011) comparing ion channel variant profiles of unaffected individuals to those with sporadic idiopathic epilepsy from targeted exome sequencing; rare missense variants were prevalent in both groups at similar complexity, demonstrating that even deleterious ion channel variants confer uncertain risk to an individual depending on the other variants with which they are combined [168]. In fact Hiekkala et al. have hypothesized that HM may not be a true monogenetic disease, but that it may reflect an extreme phenotype in the MA spectrum where rare and/or multiple common variants contribute to the disease outcome [30].

#### Assigning function to potential HM and migraine-causing variants

Determining the biological effect of variants on protein function is a major limitation in medical genetics. As NGS techniques reveal many more variants, particularly if HM is highly genetically heterogeneous, it will be necessary to improve functional testing pipelines to filter those likely to be pathogenic. Public databases which provide variant frequency (e.g. dbSNP, Genome Aggregation Database [169]) and previously reported pathogenicity information (e.g. ClinVar [170], Leiden Open Variation Databases), and in silico bioinformatics tools which predict functional consequences (e.g. SIFT [171], Polyphen2 [172], and MutationTaster) are useful in prioritising lists of candidate variants by providing first assessments of pathogenicity [173, 174, 175]. In silico methods to predict the impact of regulatory variants are also being developed [176, 177]. In addition to in silico analysis, functional assays are necessary to provide further evidence for pathogenicity, or otherwise, for prioritised variants, and to explore molecular mechanisms. Testing exogenous DNA constructs with engineered variants in cell and animal models can be complimented with genome-editing technologies, particularly the clustered regularly interspaced short palindromic repeats (CRISPR)-Cas9 system, which allows more refined and faster generation of knock-out or knock-in lines [178]. Coupled with induced pluripotent stem cells (iPSCs), which are able to be differentiated into various neuronal cell types [179, 180], as well as brain organoids [181], variants can be functionally tested in more relevant cell models, or generated from patients so they can be studied in the context of their genomic background. A range of approaches to scale up such assays are being developed [182], e.g. deep mutational scanning, which combines large scale generation of variants with deep sequencing, is a technique allowing the effect of a combination of variants to be tested at once [183], and high throughput electrophysiology platforms are available for testing ion channel variants [184].

#### Targeting treatment to genetic diagnosis in HM-related disorders

A molecular diagnosis is likely to improve management and treatment efficiencies for neurological disorders, even if symptoms may overlap, as the specific pathway or mechanism can be targeted. E.g. Glut1 deficiency caused by *SLC2A1* mutations can be treated using a ketogenic diet and HM symptoms, if present, have been found to improve on a modified Atkins diet [131]. In HM cases with *PRRT2* mutations, some benefit has been observed with carbamazepine, the most frequently used drug in treating PKD and PKD/IC patients [185]. A range of acute and prophylactic drugs are used for HM, and some may be more effective than others depending on the nature of the causative genetic mutation [22].

### Genetics of common migraine

Monogenic migraine disorders have a large impact on the individuals and families involved, but they are rare. The majority of migraine is polygenic, i.e. it is a complex disorder in which multiple variants in genes contribute to the underlying risk, with each one usually having a relatively small effect. Disease susceptibility is further a result of interaction of these genetic variations with each other, and with environmental and lifestyle factors. Discovering loci and genes that contribute to common migraine requires different approaches to the Mendelian disorders, mainly based on finding differences in allele frequencies of genetic variants linked to genes, between cohorts of migraine cases and non-migraine controls, composed of unrelated individuals. Common genetic variation largely comprises of SNPs, small insertions or deletions, short tandem repeats, and copy number variants. Most effort in identifying variants that influence traits and disorders, including migraine, has been focussed on the SNPs that confer an increased or decreased risk of migraine. These studies are demanding as, although each variant may contribute to migraine susceptibility, it is neither necessary, nor sufficient, to cause it. Effect sizes for most loci are generally small (allelic odds ratio of 1.03–1.28), requiring genotyping of large numbers of individuals to robustly obtain results that pass significance thresholds [162]. Significant differences in allele frequencies of a SNP does not necessarily mean that the SNP is itself a susceptibility factor, but that a causal variant may be in linkage disequilibrium (LD) with it. Linking the associated polymorphism to the variant that elicits the effect, or even to the gene affected, is often challenging.

### Association studies of polymorphisms in migraine candidate genes

For many years, association studies of SNPs in and around hypothesis-driven candidate genes was the main approach used to investigate genes thought to be involved in migraine. Studies generally genotyped either known functional variants, or tagging SNPs across gene loci selected from biological pathways thought to be relevant, e.g. neurological, vascular, hormonal, and inflammatory pathways [186]. Association studies of close to 200 polymorphisms in ~ 100 genes have been published for migraine [187], although subsequent and replication studies often reported conflicting results. The occurrence of false positive results in case-control study designs may be due to small sample sizes, lack of consideration for LD blocks, inadequate correction for multiple testing and phenotyping issues [40]. The C667T variant (rs1801133) in the 5,10-methylenetetrahydrofolate reductase gene (*MTHFR*), encoding a key enzyme in the folate pathway, results in an alanine to valine substitution in the catalytic domain, which reduces its activity by ~ 50% [188]. *MTHFR* C667T has been one of the most extensively studied polymorphisms in migraine; some meta-analyses report association of the T-allele with MA, but not MO [189–192], however, this has not been supported by other meta-analyses [193, 194]. Furthermore, a systematic re-evaluation of the most promising candidate gene SNPs, including *MTHFR* C667T, and others previously found to be positively associated with migraine, showed no clear evidence for involvement in migraine using International Headache Genetics Consortium (IGHC) GWAS data for 5175 clinic-based migraineurs and 13,972 controls [195]. Population stratification, where a significant association may be due to the underlying structure of the population irrespective of disease status, can contribute to biased or conflicting results in case-control studies [196]. Genetic background and population-specific risk factors may also lead to divergent findings. One *MTHFR* C667T meta-analysis reported association with migraine and MA of the T-allele, particularly in populations belonging to Asian ancestry [192].

### Genome-wide association studies (GWAS) for migraine

Hypothesis-free GWAS present a more unbiased method to identify SNPs, and potentially genes, robustly involved in migraine to gain insights into its pathways and pathophysiology. SNP arrays have enabled the simultaneous genotyping of hundreds of thousands to millions of SNPs in a sample, essentially allowing the entire genome to be scanned. Genotyped SNPs serve as a proxy for any SNPs that are in strong LD, which are tested for association with the trait in question. A number of migraine GWAS have been performed, including five major studies [53, 197–200], with the most recent meta-analysis bringing the number of associated SNPs to 44 that mapped to 38 independent genomic loci [53]. Earlier GWAS identified migraine susceptibility SNPs nearby genes with mainly putative or known neuronal functions, including *MTDH*, *PRDM16*, *TPRM8* and *LRP1* [197, 198]. LRP1 has been shown to exert regulatory effects on a number of correlated cellular events including amyloid precursor protein metabolism, kinase dependent intracellular signalling, neuronal calcium signalling and modulation of synaptic transmission through the N-methyl-D-aspartate glutamate receptors via regulating the cellular distribution of GluA1 receptors on neurons [201–203]. *TPRM8* encodes for a receptor-activated non-selective cation channel activated by cold environmental temperatures and is related to pain sensor channels [204]. PRDM16 plays roles in leukaemogenesis, palatogenesis, and brown fat cell differentiation from skeletal muscle [205], but also promotes stem cell maintenance in fetal hematopoietic and nervous systems and adult neural stem cell maintenance, neurogenesis, and ependymal cell differentiation, partly via modulating oxidative stress [206, 207].

A GWAS by Freilinger et al. (2012) had revealed that, in addition to genes involved in synapse and neuronal function and differentiation (*MEF2D* and *ASTN2*), genes with vascular functions (*TGFBR2*, *PHACTR1*) were also likely to be important in migraine susceptibility [199]. For example, *TGFBR2* encodes part of the receptor complex which transduces TGF-β signalling and regulates both synaptic and endothelial functions [208, 209]. The GWAS meta-analyses of Antilla et al. (2013) and Gormley et al. (2016), with expanded sample sizes, reiterated this fact with the discovery of further loci near genes with neuronal functions, but also many more gene loci related to functions in vascular and smooth muscle tissues, underlining their contribution to migraine pathophysiology [53, 161]. The most recent meta-analysis by Gormley et al. (2016) combined 22 GWA studies from the International Headache Genetics Consortium (IGHC), comprised 59,674 migraine cases from clinic- and population-based collections, as well as samples obtained by partnerships with the commercial entities 23andMe and deCODE, and 316,078 controls [53]. This study brought the number of SNPs significantly associated with migraine to 44 independent SNPs at 38 distinct genomic loci, and included the majority of GWAS loci previously reported, as well as an additional 28 novel loci, including the first on the X chromosome (Near *MED14*-*USP9X*). Database annotations and relevant literature for the genes in LD with the SNPs have been reviewed by Gormley et al. (supplementary tables) [53] and Sutherland et al. (table) [93].

The meta-analysis by Gormley et al. confirmed the single most significant SNP as rs11172113 in the *LRP1* gene locus, and that the genes prioritised as likely candidates at many of the loci have known or putative roles in vascular function (e.g. *LRP1*, *PRDM16*, *ECM1*, *MEF2D*, *TGFBR2*, *ARHGEF26*, *REST*, *PHACTR1*, *NOTCH4*, *FHL5*, *GJA1*, *HEY2*, *NRP1*, *PLCE1*, *HTRA1*, *YAP1*, *FGF6*, *ZCCHC14*, *JAG1*, and *CCM2L*) and the expression of many of these is highly enriched in vascular tissues [53, 162]. Furthermore, consistent with the mechanisms that have been elucidated from FHM, two of the loci are near ion channels genes, *TPRM8* and *KCNK5*, the latter a member of the same family as *KCNK18*. Three additional loci are linked to the *SLC24A3*, *ITPK1* and *GJA1* genes, which all have a function in cellular ion homeostasis. More unexpectedly, many genes that contribute to migraine susceptibility are involved in metal ion homeostasis according to Gene Ontology (GO) terms (*PRDM16*, *TGFBR2*, *REST*, *FHL5*, *NRP1*, *MMPED2*, *LRP1*, *ZCCHC14*, *RNF213*, *JAG1*, *SLC24A3*) suggesting the importance of these pathways in migraine pathophysiology [162]. Metal ions (including Fe^2+^, Cu^2+^, Co^2+^, Mn^2+^, Ca^2+^, Na^+^, and Zn^2+^) are essential in many metabolic processes and their transport and storage into cellular compartments is highly regulated [210]. How these processes might be contribute to migraine remains to be fully elucidated, however, it is known for example, that synaptic zinc is a potent modulator of neurotransmission [211].

It should be noted that many of the loci have both neuronal and vascular functions, and/or roles in multiple pathways [53, 93, 162]. For example, *NRP1* encodes neuropilin 1, a cell surface glycoprotein which mediates axon guidance and adhesion during GABAergic synapse formation in developing nervous system [212], but is also involved in vascular patterning and cardiovascular system development as a receptor for the vascular guidance molecule semaphoring 3d [213]. Furthermore, there is some overlap in pathways between monogenic migraine genes and GWAS loci. In common with the monogenic FHM and MA forms caused by ion channel gene mutations, some ion channel gene loci are implicated in polygenic migraine. Similarly, genes of the Notch signalling pathway are involved in both the monogenic migraine-related cerebrovascular disorder CADASIL (caused by pathogenic *NOTCH3* variants) and common migraine, with GWAS loci identified near both the *NOTCH4* receptor gene, and *JAG1*, which encodes Jagged1, a ligand of multiple Notch receptors.

#### Fine mapping and functional analysis of migraine associated SNPs

Analyses of the genes in the vicinity of GWAS loci has suggested the types of gene function and pathways that may be involved in migraine, however, it is important to remember that for the majority of loci, the gene that is actually influenced by the SNP remains unknown. SNPs affect the diversity of human traits/diseases via various mechanisms: changing encoded amino acids of a protein (non-synonymous) may affect its function or localisation; and SNPs that are either silent (synonymous), or more commonly, in noncoding regions, may affect gene expression levels via messenger RNA (mRNA) conformation and stability, subcellular localization, or its promoter/enhancer activity. Making the leap from associated SNPs to causal genes, and then to functional mechanisms, still presents a formidable task in the interpretation of GWAS.

Methods have been developed to fine-map GWAS loci, combining statistical and functional evidence [214, 215]. Firstly, association-test statistics can be combined with LD information to prioritise a credible set of SNPs likely to contain the causal disease-associated SNP. As susceptibility SNPs often lie in introns or intergenic regions, the next hurdle is to identify which gene is affected (not necessarily the nearest), by connecting the variants with genes by a range of methods and resources, complementing functional annotation with information from projects such as ENCyclopedia of DNA Elements (ENCODE), NIH Roadmap Epigenomics, and FANTOM5, which have characterized regulatory regions and expression quantitative trait loci (eQTL) [162, 214]. Once putative variants and genes have been pinpointed via in silico analysis, further functional experiments are required to confirm and understand molecular mechanisms. This process is illustrated by investigations into rs9349379 in intron 3 of the *PHACTR1* gene, which has been identified as a causal susceptibility SNP in a range of vascular disorders including migraine [216]. From epigenomic data from human tissues, Gupta et al. (2017) identified an enhancer signature over rs9349379 in aorta suggesting a vascular regulatory function; then using CRISPR-edited stem cell-derived endothelial cells they demonstrated that the SNP actually regulates expression of the endothelin 1 gene (*EDN1*), located 600 kb upstream of *PHACTR1* [216]. *EDN1* encodes a 21 amino acid peptide that, along with its receptor, promotes vasoconstriction, vascular smooth muscle cell proliferation, extracellular matrix production, and fibrosis; these factors would contribute to the increased risk of coronary artery disease and decreased risk of cervical artery dissection, fibromuscular dysplasia and migraine, conferred by the SNP [216]. This work underlines the importance of functional assays in cellular and animal models in further characterisation of migraine GWAS signals.

In another effort to refine GWAS loci, Hannon et al. applied summary-data-based Mendelian randomization (SMR) to large DNA methylation quantitative trait locus (mQTL) datasets generated from blood and fetal brain to prioritize genes for > 40 complex traits with well-powered GWAS data, including migraine [217]. Using this approach they showed that, with respect to the *HEY2*-*NOCA7* GWAS signal identified by Gormley et al. [53], whole blood and fetal brain have a mQTL profile highly comparable to that of the migraine GWAS, which implicated *HEY2* in migraine. These results are consistent with genetic signals influencing DNA methylation in both tissues and migraine, and shows utility of this approach in prioritizing specific genes within genomic regions identified by GWAS [217]. The expansion of resources with gene expression and epigenetic data in tissues relevant to migraine-related pathophysiology will be critical to advancing these types of studies. Recent studies have used gene expression datasets (including single cell analysis) to begin to link genetic loci to their expression in migraine-relevant brain tissues and cell types [218–220].

#### Migraine susceptibility loci in migraine sub-types

There has been some discussion about whether MO and MA are different entities or part of a disease spectrum [221–223]. Subtype analysis in high-powered GWAS with large samples sizes may reveal whether particular genes may contribute to phenotypic consequences. Most of the migraine loci identified by Gormley et al., (2016) were implicated in both MO and MA, although seven genomic loci (near *TSPAN2*, *TRPM8*, *PHACTR1*, *FHL5*, *ASTN2*, near *FGF6* and *LRP1*) were significantly associated with the MO subtype [53]. None were significant for MA, likely reflecting the smaller sample size. Some genetic loci may be selectively associated with particular features (e.g. pain character, duration, frequency, nausea, photophobia and triggers) of the migraine attack [224, 225]. Menstrual migraine affects a subset of female MO sufferers; replication of migraine GWAS loci in a menstrual migraine case-control cohort suggested a particular role for *NRP1* in this subgroup [226]. However, the small sample sizes often make it difficult to obtain robust associations for such specific phenotypes. Nevertheless, it will be interesting to identify genes that might be involved in specific aspects of migraine.

#### Shared genetic factors with other disorders

A wider view is also informative and can be used to explore the etiology of related and comorbid traits. A GWAS of broadly defined headache using the UK Biobank data found significant associations at 28 loci, of which 14 overlapped with migraine, including the rs11172113 in the *LRP1* as the top SNP [227]. Some migraine-associated genes and SNPs have more systemic effects and are involved in a wide range of disorders. A large analysis of shared heritability between common brain disorders found that while most psychiatric and neurologic disorders share relatively little common genetic risk, suggesting largely independent etiological pathways, migraine appears to share some genetic architecture with psychiatric disorders, including attention deficit hyperactivity disorder (ADHD), Tourette’s syndrome, and major depressive disorder [228]. This, together with genetic correlations with other neurological (epilepsy) and vascular disorders (stroke, coronary artery disease), is consistent with comorbidities that have been documented for migraine and suggests they are underpinned by shared genetic factors [228–233]. Similarly, the monogenic migraine disorders show comorbidity with epilepsy, depression, vascular and sleep disorders [54, 145, 234, 235]. Understanding these relationships can impact the management and treatment of conditions with overlapping etiologies [235, 236].

#### Migraine susceptibility loci in migraine in specific populations

As the large migraine GWAS have been performed in predominantly Caucasian populations of European heritage, questions remain as to whether the genes and SNPs identified are relevant to other ethnicities, and if there are population-specific genes and polymorphisms. One way to address the former is to test whether there is replication of association of the GWAS SNPs in a particular population. A number of studies have taken this approach, both in specific European cohorts, as well as North Indian and Han Chinese. For example, association of the minor C allele for the *PRDM16* polymorphism rs2651899 was replicated in Swedish [237], Spanish [238] and Han Chinese cohorts [239, 240], while rs2651899 and *LRP1* rs11172113 showed a protective effect on migraine susceptibility in a North Indian population [241]. Polymorphisms rs4379368 (Succinyl-CoA:Glutarate-CoA Transferase gene locus, *C7orf10*) and rs13208321 (*FHL5*) showed some replication in a cohort of the Chinese She people [242]. However, GWAS conducted in specific ethnic populations will determine whether the genetic contributions to migraine vary, and identify migraine susceptibility loci which may be particular to different groups. While still limited, and with relatively small sample sizes, GWAS have been performed in Norfolk Islander, Taiwanese Han Chinese and African American pediatric cohorts [243–245]. The Norfolk Island genetic isolate is a unique admixed Polynesian-Caucasian population with a high prevalence of migraine (25%). A GWAS for migraine revealed a number of loci of suggestive significance near neurotransmitter-related genes [245]. A GWAS in Taiwanese Han Chinese identified two novel migraine susceptibility SNPs: rs655484 in *DLG2*, a gene involved in glutamatergic neurotransmission; and rs3781545 in *GFRA1*, which encodes a receptor for glial cell line-derived neurotrophic factor (GDNF) in trigeminal neurons [243]. The GWAS in American African children found association of migraine with SNPs, including rs72793414, which were strongly correlated with the mRNA expression levels of *NMUR2*, encoding the G protein-coupled receptor of the CNS neuropeptide neuromedin-U [244].

### Genetic risk scores (GRS) and applications for migraine

Due to low effect sizes that the majority of variants have on associated traits, the genotype at an individual SNP does not have particular diagnostic or prognostic value in common migraine. However, calculating a genetic risk score (GRS) or polygenic risk score (PRS), which assesses the additive effect of many associated SNPs from sufficiently powered studies, may have utility in disease prediction [246]. With the availability of increasingly large GWAS data sets for migraine, GRS may be applied to: investigating migraine subtypes and endophenotypes, understanding migraine pleiotropy and co-morbidites, disease and phenotype prediction, and for assessing pharmocogenetic effects for personalised medicine [247]. Higher GRS have been correlated with migraine diagnosis in specific cohorts [226, 248], as well as migraine severity, and in cases where migraine is aggregated in families suggesting this results from a higher common variant burden [225, 249]. One particular use of GRS may be in understanding drug reactions and efficacy of therapies. Studies to predict response and efficacy of treatment with triptans in migraineurs have used this approach [250, 251]. While sensitivity and specificity are still relatively low, the diagnostic value of GRS will improve with the discovery of more SNPs. With respect to drug and treatment responses, this would include variants that affect the genes targeted by drugs, but also those involved in drug transport and metabolism [252, 253].

### Powering up GWAS and genomic sequencing

It is likely that common variants will not completely explain common migraine, but that rare private variants (with small to medium effects) will contribute as well. This has been demonstrated by the well-studied trait of adult human height, which has a strong genetic component (estimated heritability up to 80%). Meta-analysis of multiple GWAS with a combined sample size of > 250,000 individuals has yielded ~ 700 common SNPs clustered in 423 independent loci that contribute to height [254]. These, however, still only capture ~ 20% of the heritability. Compound heterozygote-like SNP interactions may further contribute to phenotypic variance [255]. Furthermore, using ExomeChips, Marouli et al. identified a further 83 coding variants with lower minor-allele frequencies (in the range of 0.1–4.8%) associated with height [256]. However, in addition to further scaling up of sample sizes, ultimately WGS will be required to truly discover all of the DNA sequence contribution to the trait. For migraine, sample sizes are still relatively small compared to the studies that have been done for traits like height and obesity, i.e. > 500,000 individuals including 170,000 Japanese [257, 258]. It is likely that more migraine-related loci will be discovered as sample numbers increase in migraine GWAS using SNP-chips (including from various ethnicities), and the effect of rare variants identified from exonic and genomic sequencing becomes clearer. Integrating genetic and other genomic information, such as transcriptional and epigenetic data, will deepen understanding of the important tissues and pathways in migraine [218, 259]. 

## Conclusions

**Fig. 1:**
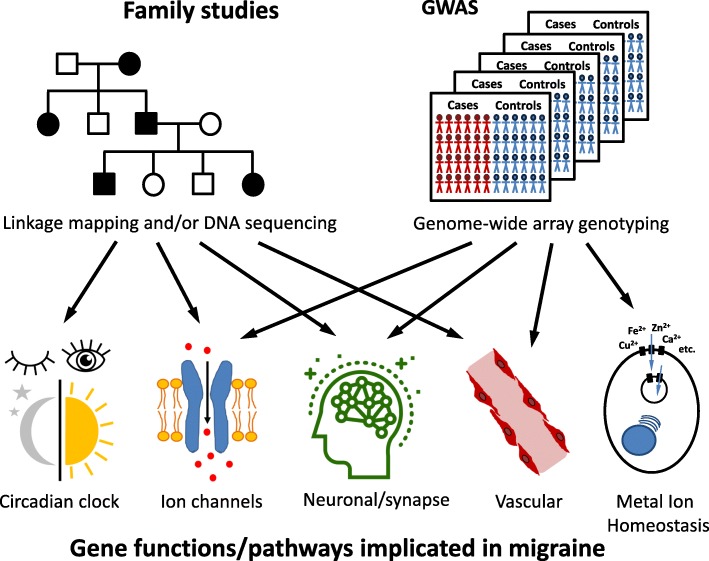
Approaches to identifying the genes involved in migraine and their functions and putative pathways

Migraine is a multifactorial disorder with genetics playing an important role in the susceptibility, and symptomology, as well as comorbidity with other traits and conditions. Investigation of the genetic factors involved in migraine have used family studies for the rare, Mendelian forms of migraine, as well as GWAS in case-control cohorts for the common polygenic form of migraine, for gene discovery and further understanding of the pathways and basic biology of the disorder (Fig. 1). For monogenic migraine, mapping of loci in family pedigrees, coupled with genomic sequencing to find variants, led to the discovery of the main FHM genes, *CACNA1A*, *ATP1A2* and *SCN1A*. Knowledge of their roles as ion channels and in ion transport, along with functional experiments in cellular and animal models, has contributed to uncovering how their dysfunction may lead to cortical hyperexcitability and migraine. Mutations in other genes can also cause HM, and it is likely that pathogenic variants in more genes will be discovered, with NGS technologies (WES and WGS) accelerating this research. With respect to the common polygenic forms of migraine, GWAS analyses using high-throughput SNP genotyping arrays has revealed many variants around genes with roles in neurological and vascular pathways in migraine. With increasing sample sizes more susceptibility loci are likely to be found, some of which may contribute to specific migraine subtypes or symptoms. Moving from finding a risk SNP, to the gene, to the molecular mechanism, still remains challenging, but developments around methods for functional studies, including iPSC models and genome-editing, will facilitate such research.

Genetics has further emphasized the complexity of migraine disorders, but it is an exciting time to be working in the field of migraine biology, with the end game – to better diagnose, manage and treat migraine sufferers.

## Data Availability

Not applicable.

## Abbreviations

BFIE: Benign familial infantile epilepsy
CADASIL: Cerebral Autosomal Dominant Arteriopathy with Subcortical Infarcts and Leukoencephalopathy
CGRP: Calcitonin gene-related peptide
CNS: Central nervous system
CRISPR: Clustered regularly interspaced short palindromic repeats
dbSNP: Single Nucleotide Polymorphism Database
DMRs: Differentially methylated regions
EA: Episodic ataxia
ENCODE: ENCyclopedia of DNA Elements
ExAC: Exome Aggregation Consortium
FANTOM: Functional Annotation of the Mammalian Genome
FASPS: Familial advanced sleep phase syndrome
FHM: Familial hemiplegic migraine
GABA: Gamma-Aminobutyric acid
GDNF: Glial cell line-derived neurotrophic factor
gnomAD: Genome Aggregation Database
GRS: Genetic risk score
GTex: Gene-tissue expression project
GWAS: Genome-wide association study
HM: Hemiplegic migraine
ICCA: Infantile convulsions and choreoarthetosis
IHGC: International Headache Genetics Consortium
iPSCs: Induced pluripotent stem cells
KI: Knock-in
KO: Knock-out
LD: Linkage disequilibrium
LOVD: Leiden Open Variation Databases
MA: Migraine with aura
MIM: Mendelian Inheritance in Man
MO: Migraine without aura
mQTL: Methylation quantitative trait locus
mRNA: Messenger RNA
NGS: Next-generation sequencing
NIH: National Institute of Health
PACAP: Pituitary adenylate cyclase-activating peptide
PED: Paroxysmal exercise-induced dyskinesia
PKD: Paroxysmal kinesigenic dyskinesia
PNKD: Paroxysmal nonkinesigenic dyskinesia
PRS: Polygenic risk score
ROSAH: Retinal dystrophy, optic nerve edema, splenomegaly, anhidrosis and migraine headache
RTA: Renal tubular acidosis
SHM: Sporadic hemiplegic migraine
SMR: Summary-data-based Mendelian randomization
SNARE: SNAP Receptor
WES: Whole exome sequencing
WGS: Whole genome sequencing
